# Supplementary material for: Development and Validation of the Mexican Public Open Spaces Tool (MexPOS)
Source: Int J Environ Res Public Health. 2022 Jul 5;19(13):8198. doi: 10.3390/ijerph19138198 (PMC9266626; doi:10.3390/ijerph19138198)
Supplement: Supplementary file 1 [file ijerph-19-08198-s001.zip › File S1. Final questionnaire-Spanish.pdf]

CUESTIONARIO DE EVALUACIÓN, MEDIANTE OBSERVACIÓN DIRECTA, DE LAS CARACTERÍSTICAS AMBIENTALES DEL PARQUE.

**Check list:**	Si	no
ANTES DE LLEGAR AL PARQUE…		
¿Leíste el manual?		
¿Llenaste la ficha general (sección 1)?		
ANTES DE LLENAR EL CUESTIONARIO…		
¿Caminaste alrededor del parque antes de llenar éste cuestionario?		
ANTES DE DAR POR FINALIZADA LA CAPTURA DEL CUESTIONARIO…		
¿Llenaste todos las secciones del cuestionario?		
¿Revisaste todas las respuestas?		
¿En caso de tener error en alguna respuesta, corregiste de forma correcta?		

¿Qué hacer en caso de error?

Marque con una "X" la respuesta correcta, en caso de error, rellenar el cuadro y marcar con una "X" la respuesta correcta.

[x] ➡ ■ Error

[x] Corrección

Encuestador: ☐☐☐

Código del parque: ☐☐☐

1. Fecha de la observación | d | d | / | m | m | / | a | a | a | a |

2. Temperatura ☐☐ °C

   (ingresa a: https://weather.com/esUS/tiempo/hoy/l/Ciudad+de+Mexico+Mxico+MXDF0132:1:MX)

3. Clima
   - 1 ☐ Soleado
   - 2 ☐ Parcialmente soleado
   - 3 ☐ Parcialmente nublado
   - 4 ☐ Nublado
   - 5 ☐ Lluvioso

   (ingresa a: https://weather.com/esUS/tiempo/hoy/l/Ciudad+de+Mexico+Mxico+MXDF0132:1:MX)

4. Calidad del aire

				IMECA

(ingresa a: http://aqicn.org/map/world/es/#@g/12.0511/-82.1997/5z)

5. Hora inicio de captura

		:			Am	Pm

6. Hora de término

		:			Am	Pm

7. Momento del día de captura

☐ 1.Día ☐ 2.Noche

1. FICHA GENERAL

Llenar éste apartado ANTES de llegar al parque.

1.1 Nombre del parque: ____

1.2 Ubicación: ____

1.3 Tipología:

1 ☐ Parque Metropolitano
2 ☐ Parque Local
3 ☐ Parque Barrial
4 ☐ Parque de Bolsillo
5 ☐ Parque Remanente
6 ☐ Glorieta
7 ☐ Jardín
8 ☐ Plaza
9 ☐ Alameda

2. ASPECTOS GENERALES DEL PARQUE
a. Actividades dentro del parque.

Ésta sección recolecta información sobre todas las áreas del parque donde se puede realizar actividad física.

2a.1. ¿Qué áreas de actividad hay en el parque? *Indica todas las que correspondan.*

	Sí	No	
1	1 ☐	2 ☐	Cancha de volibol
2	1 ☐	2 ☐	Cancha de basquetbol
3	1 ☐	2 ☐	Cancha de frontón
4	1 ☐	2 ☐	Cancha de tenis
5	1 ☐	2 ☐	Campo de fútbol americano
6	1 ☐	2 ☐	Campo de fútbol soccer
7	1 ☐	2 ☐	Otros campos, ¿cuál? ____________________(2a.1_7a)
8	1 ☐	2 ☐	Senderos peatonales
9	1 ☐	2 ☐	Trotapista
10	1 ☐	2 ☐	Ciclopista
11	1 ☐	2 ☐	Parques infantiles
12	1 ☐	2 ☐	Áreas verdes
13	1 ☐	2 ☐	Jardines botánicos
14	1 ☐	2 ☐	Área de comer
15	1 ☐	2 ☐	Área para sentarse
16	1 ☐	2 ☐	Teatro al aire libre
17	1 ☐	2 ☐	Cancha de fútbol rápido
18	1 ☐	2 ☐	Cancha de fútbol 7
19	1 ☐	2 ☐	Cancha combinada de fútbol y básquetbol
20	1 ☐	2 ☐	Barras
21	1 ☐	2 ☐	Skatepark
22	1 ☐	2 ☐	Otro, ¿cuál? __________________________________(2a.1_22.1)
22a	1 ☐	2 ☐	Otro, ¿cuál? __________________________________(2a.1_22a.1)
22b	1 ☐	2 ☐	Otro, ¿cuál? __________________________________(2a.1_22b.1)

2a.2. Indica TODAS las áreas que son usadas por los usuarios.

1	☐	Cancha de volibol
2	☐	Cancha de basquetbol
3	☐	Cancha de frontón
4	☐	Cancha de tenis
5	☐	Campo de fútbol americano
6	☐	Campo de fútbol soccer
7	☐	Otros campos, ¿cuál? ______________ (2a.2_6a)
8	☐	Senderos peatonales
9	☐	Trotapista
10	☐	Ciclopista
11	☐	Parques infantiles
12	☐	Áreas verdes
13	☐	Jardines botánicos
14	☐	Área de comer
15	☐	Área para sentarse
16	☐	Teatro al aire libre
17	☐	Cancha de fútbol rápido
18	☐	Cancha de fútbol 7
19	☐	Cancha combinada de fútbol y básquetbol
20	☐	Barras
21	☐	Skatepark
22	☐	Otro, ¿cuál? ____(2a.2_22.2)
22a	☐	Otro, ¿cuál? ____(2a.2_22a.2)
22b	☐	Otro, ¿cuál? ____(2a.2_22b.2)

2a.3. ¿Qué áreas se encuentran en BUENA condición? *Califica TODAS las áreas.*

	Áreas	1.Totalmente de acuerdo	2. De acuerdo	3.Ni de acuerdo ni en desacuerdo	4.En desacuerdo	5.Totalmente en desacuerdo	6. No aplica
1	Cancha de volibol						
2	Cancha de basquetbol						
3	Cancha de frontón						
4	Cancha de tenis						
5	Campo de fútbol americano						
6	Campo de fútbol soccer						
7	Otros campos, ¿cuál? _______________(2a.3_7)						
8	Senderos peatonales						
9	Trotapista						
10	Ciclopista						
11	Parques infantiles						
12	Áreas verdes						
13	Jardines botánicos						
14	Área de comer						
15	Área para sentarse						
16	Teatro al aire libre						
17	Cancha de fútbol rápido						
18	Cacha de futbol 7						
19	Cancha combinada de fútbol y básquetbol						
20	Barrar						
21	Skatepark						
22	Otro, ¿cuál? _____________________(2a.3_22)						
22a	Otro, ¿cuál? _____________________(2a.3_22a)						
22b	Otro, ¿cuál? _____________________(2a.3_22b)						

2a.4. ¿Se realizan actividades diferentes para lo que están destinadas las áreas?

1 ☐ Sí
2 ☐ No ⇨ *Pasa a sección "información y comunicación)*

2a.5. ¿Qué actividades observas que realizan en estos espacios? *Si los espacios no son utilizados para las actividades destinadas anotar.*

1. Canchas (fútbol rápido, basquetbol) ____ (2a.5.1)
2. Campos ____ (2a.5.2)
3. Áreas verdes ____ (2a.5.3)

b. Información y comunicación.

Está sección pretende describir si el parque cuenta con mapas, normas de comportamiento y equipos rentables.

2b.1. ¿Existe señalización o punto de referencia que indique el nombre del parque?

1 ☐ Sí, ¿cuál? (ejemplo: cartel formal, cartel informal) ___________________________(2b.1.1.a)
2 ☐ No

2b.2. ¿Existe un mapa general del parque?

1 ☐ Sí
2 ☐ No

2b.3. ¿Existe algún cartel o señal que indique las normas de comportamiento en el parque (por ejemplo: NO escupir, fumar, mascotas, restricción a ciertas zonas, etc.)?

1 ☐ Sí, ¿cuál? __________________________________(2b.3.a)
2 ☐ No

2b.4. ¿Existe algún cartel o señal que indique quiénes son los encargados del parque?

1 ☐ Sí
2 ☐ No

2b.5. ¿Existe algún cartel o señal que indique si el parque cuenta con equipos rentables (por ejemplo: lanchas, bicicletas, carritos, etc.)?

1 ☐ Sí, especifique cuál (es) ____(2b.5.a)
2 ☐ No

2b.6. ¿Existe algún cartel o señal informando sobre algún evento en el parque?

1 ☐ Sí, ¿qué tipo de eventos? ____(2b.6.1)
2 ☐ No

2c. Estética

Esta sección pretende conocer la estética y ambiente del parque, por ejemplo: si es atractivo visualmente, sonidos y olores.

2c.1. De acuerdo a lo observado hoy, ¿El parque es atractivo?

1 ☐ Totalmente de acuerdo
2 ☐ De acuerdo
3 ☐ Ni de acuerdo ni en desacuerdo
4 ☐ En desacuerdo
5 ☐ Totalmente en desacuerdo

2c.2. ¿La condición del paisaje es adecuada?

1 ☐ Totalmente de acuerdo
2 ☐ De acuerdo
3 ☐ Ni de acuerdo ni en desacuerdo
4 ☐ En desacuerdo
5 ☐ Totalmente en desacuerdo

2c.3. ¿Hay grafiti?

1 ☐ Sí
2 ☐ No ⇨ *Pasa a 2c.5*

2c.4. ¿Cuánto grafiti es visible?

1 ☐ Mucho
2 ☐ Algo
3 ☐ Poco
4 ☐ Nada

2c.5. ¿Observas exceso de basura en el parque?

1 ☐ Sí, ¿dónde? ____(2c.5.a)
2 ☐ No

2c.6. ¿Observas basura de excremento de animales en el parque? 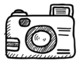

1 ☐ Sí, ¿dónde? ____(2c.6.1)
2 ☐ No
3 ☐ No aplica

2c.7. ¿Existe alguna fuente de sombra?

1 ☐ Sí
2 ☐ No ⇨ **Pasa a 2c.9**

2c.7b. ¿Cuáles son las fuentes de sombra y en qué cantidad se encuentran? *Indica todas las que correspondan*

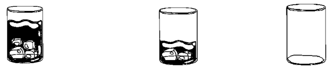

			1.	2.	3.
1	☐	Árboles, ¿cuánto? (2c.7b_1a)	Mucho	Poco	Nada
2	☐	Edificios, ¿cuánto? (2c.7b_2a)	Mucho	Poco	Nada
3	☐	Refugios, ¿cuánto? (2c.7b_3a)	Mucho	Poco	Nada
4	☐	Carpas, ¿cuánto? (2c.7b_4a)	Mucho	Poco	Nada
5	☐	Otro, ¿cuál?,_____________(2c.7_5a)	Mucho	Poco	Nada

Refugio se refiere a elementos arquitectónicos como son pérgolas, techumbres y quioscos que sirven para asilarse del sol y/o la lluvia.

2c.8. En total, ¿cuánto del parque podría estar sombreado? *<u>*Nota: llenar al final del recorrido</u>.*

0 ☐ 0
1 ☐ 1-25%
2 ☐ 26-75%
3 ☐ 76-100%

2c.9. ¿Qué sonidos escuchas? *Indica todas las que correspondan.*

1 ☐ Agua, ¿dónde? ____ (2c.9_1a)
2 ☐ Pájaro
3 ☐ Tráfico, ¿dónde? _______________________________________ (2c.9_3a)

4 ☐ Construcción, ¿dónde? ________________________________ (2c.9_4a)
5 ☐ Voces, ¿dónde? _____________________________________ (2c.9_5a)
6 ☐ Música, ¿dónde? ____________________________________ (2c.9_6a)
7 ☐ Otro, ¿cuál? ____ (2c.9_7a)

2c.10. De acuerdo al día de hoy, ¿el olor en general del parque es agradable?

1 ☐ Totalmente de acuerdo
2 ☐ De acuerdo
3 ☐ Ni de acuerdo ni en desacuerdo
4 ☐ En desacuerdo
5 ☐ Totalmente en desacuerdo

2c.11. ¿En general, el parque cuenta con BUEN mantenimiento?

1 ☐ Sí
2 ☐ No

2c.12. ¿El parque cuenta con características históricas, educativas o artísticas? Por ejemplo: monumentos, visualización de la naturaleza, estatuas, esculturas, fuentes, etc.)

1 ☐ Sí, especificar ________________________________(2c.12.a)
2 ☐ No

2c.13. ¿En general, cuánto porcentaje del parque es área enselvada? Por ejemplo: bosques o árboles densos.

0 ☐ 0
1 ☐ 1-25%
2 ☐ 26-75%
3 ☐ 76-100%

2c.14. ¿El parque cuenta con elementos de agua como: lagos, arroyos o estanques?

1 ☐ Sí, ¿dónde? ________________________________(2c.14a)
2 ☐ No

3. ACCESIBILIDAD

Está sección pretende identificar si el parque está abierto a la población en general, si tiene conexión con medios de transporte, así como estacionamientos.

3.1. ¿La apertura del parque u operación del parque depende de un conserje/administrador?

1 ☐ Sí
2 ☐ No

3.2. ¿El parque está bardeado o enrejado?

1 ☐ Sí, porcentaje ______________________(3.2a)
2 ☐ No

3.3. ¿Existe algún anuncio especificando las horas de operación del parque? 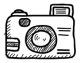

1 ☐ Sí
2 ☐ No

3.4. ¿Cuántas entradas tiene el parque para los usuarios?

1 ☐ Solo 1
2 ☐ 2-5
3 ☐ Más de 5
4 ☐ Es parque abierto

3.5. ¿Todas las áreas del parque se encuentran abiertas para el público en general?

1 ☐ Sí ⇨ *Pasa a 3.9*
2 ☐ No

3.6. ¿Enlista las áreas que NO son de acceso al público en general?

____
____
____
____

3.7. ¿Se requiere una membresía o cuota para acceder a estas áreas?

1 ☐ Sí
2 ☐ No ⇨ *Pasa a 3.9*

3.8. ¿Cuál es el costo aproximado de ésta membresía/cuota?

$ ☐☐☐☐

3.9. ¿El parque cuenta con elementos que garanticen la accesibilidad universal? Por ejemplo: rampas, barandales, mapas hápticos, etc.

	Elementos	Si	No
1	Rampas	1 ☐	2 ☐
2	Barandales	1 ☐	2 ☐
3	Mapas hápticos	1 ☐	2 ☐
4	Señalización en braile	1 ☐	2 ☐
5	Otro, ¿cuál?____________________(3.9_5a)	1 ☐	2 ☐

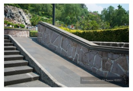
Ilustración 1 Rampas

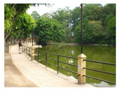
Ilustración 2 Barandales

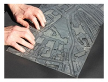
Ilustración 3 Mapas hápticos

3.10. En un radio de 100 m. ¿cuántas estaciones de transporte público existen? (estaciones de metro, paradas de autobús, etc.).

	Estaciones de transporte	Número
1	Estaciones de metro	☐☐
2	Paradas de autobús	☐☐
3	Estación de taxi	☐☐
4	Metrobus	☐☐
5	EcoBici	☐☐
6	____________________	☐☐
7	____________________	☐☐

3.11. ¿Existen paradas de transporte público a la vista desde el parque?

1 ☐ Sí
2 ☐ No

3.12. ¿El parque cuenta con estacionamiento para autos?

1 ☐ Sí
2 ☐ No ⇨ *Pasa a 3.15*

3.13. ¿Tiene algún costo el estacionamiento de autos? ¿cuál es el costo?

1 ☐ Si, costo $ ☐☐☐☐ (3.13a)
2 ☐ No

3.14. ¿Cuántos espacios hay para estacionar autos?

1 ☐ Menos de 10
2 ☐ 10-20
3 ☐ Más de 20

3.15. ¿El parque cuenta con estacionamiento para motocicletas?

1 ☐ Sí
2 ☐ No ⇨ *Pasa a 3.18*

3.16. ¿Tiene algún costo el estacionamiento de las motocicletas? ¿cuál es el costo?

1 ☐ Si, costo $ ☐☐☐☐ (3.16a)
2 ☐ No

3.17. ¿Cuántos espacios hay para estacionar motos?

1 ☐ Menos de 10
2 ☐ 10 a 20
3 ☐ Más de 20

3.18. ¿El parque cuenta con estacionamiento para bicicletas?

1 ☐ Sí
2 ☐ No ⇨ *Pasa a 3.22*

3.19. ¿Tiene algún costo el estacionamiento de las bicicletas? ¿Cuál es el costo?

1 ☐ Si, costo $ ☐☐☐☐ (3.19a)
2 ☐ No

3.20. ¿Cuántos espacios hay para estacionar bicicletas?

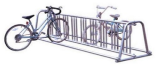

1 ☐ Menos de 10
2 ☐ 10 a 20
3 ☐ Más de 20

3.21. ¿Cuántos estantes de estacionamiento para bicicletas son funcionales?

1 ☐ Todos 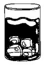
2 ☐ Algunos 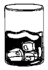
3 ☐ Pocos 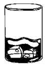
4 ☐ Ninguno 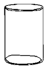

3.22. ¿Hay señalización restrictiva para estacionar la bicicleta dentro del parque?

1 ☐ Sí
2 ☐ No

3.23. ¿Hay una ruta para bicicletas que colinde con el parque?

1 ☐ Sí
2 ☐ No ⇒ *Pasa a sección 4 “Contexto inmediato”*

3.24. ¿La ruta para bicicletas está claramente señalizada (señalización horizontal o vertical)?

1 ☐ Sí, ¿cuál? ____________________(3.24a)
2 ☐ No

4. CONTEXTO INMEDIATO.

Esta sección pretende identificar el ambiente alrededor del parque. Las características de las calles, así como elementos de seguridad en las [illegible]

4.1. ¿Cuáles son los principales usos de suelo alrededor del parque? *Indica todas las que correspondan.*

	Sí		No		
1	1		2		Residencial/habitacional
2	1		2		Comercial
3	1		2		Institucional (por ejemplo escuelas) → equipamiento urbano.
4	1		2		Industrial
5	1		2		Natural
6	1		2		Otro, especificar ______________________________ (4.1.6a)

4.2. ¿Cuál de los siguientes problemas de seguridad y/o apariencia están presentes en la zona que rodea el parque? *Indica todas las que correspondan.*

	Sí		No		
1	1		2		Poca iluminación en las calles de la zona.
2	1		2		Grafiti (por ejemplo: pinturas que reducen la calidad visual del área)
3	1		2		Vandalismo (por ejemplo: señales de daños).
4	1		2		Basura.
5	1		2		Tráfico pesado.
6	1		2		Ruido excesivo.
7	1		2		Construcciones vacías o desagradables (por ejemplo: casas abandonadas).
8	1		2		Propiedades con poco mantenimiento.
9	1		2		Falta de personas en las calles.
10	1		2		Evidencia de comportamientos o personas amenazantes (por ejemplo: bandas, alcohol, uso de droga).
11	1		2		Otro, especificar __________________________________ (4.2.11)

4.3. ¿Cómo se articulan las calles con el parque? *Indica todas las que correspondan.*

	Sí		No		
1	1		2		El parque se encuentra rodeado por calles
2	1		2		El parque se encuentra atravesando la calle
3	1		2		El parque contiene dentro, una calle

4.4. Enlista las calles alrededor del parque. Para cada calle, rellena los siguientes incisos (2-9) usando la escala que aparece hasta abajo del cuadro.

1.Calle número (enlista las calles)	2. Nombre de la calle	3. Tipo de calle	4. Señales de tráfico existentes en la calle					5. ¿Los cruces son seguros en esa calle?	6. ¿Hay áreas de resguardo para el peatón?	7. ¿Cuál es el límite de velocidad?	8. ¿Hay banquetas?	9. Condición de banquetas
			Semáforo peatonal	Semáforo para autos	Cruces de cebra	Letrero de no estacionarse	Otro, ¿cuál? ____					
Números (1 al 100)	Letras (rellena)	1.Primaria<br>2.Secundaria<br>3.Terciaria	1. Si<br>2. No					1. Si<br>2. No	1. Si<br>2. No	Km/h<br>2. No cuenta con letrero	1. Si<br>2. No	1. Buena<br>2. Regular<br>3. Mala

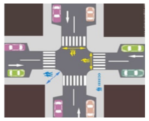
Cruce peatonal con una ampliación de acera tipo "oreja"

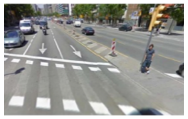
Isla peatonal

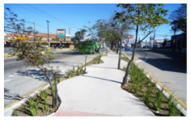
Camellón peatonal

5. CAMINOS/RUTAS INTERNAS/PISTA DENTRO DEL PARQUE

Esta sección pretende identificar la condición del camino/ruta/pista de mayor jerarquía dentro del parque, así como sus características.

5.1. ¿Existe algún camino/ruta/pista dentro del parque?

1 ☐ Sí
2 ☐ No ⟹ *Pasa a sección 6 "Seguridad e iluminación"*

5.2. Medir el ancho del camino/ruta/pista (en caso de que sean varias, seleccionar la de mayor jerarquía*)

1 ☐ <0.6 metros
2 ☐ 0.6-1.5 metros
3 ☐ Más de 1.5 metros
4 ☐ No se puede evaluar

5.3. ¿De qué material está hecha la superficie del camino/ruta/pista?

1 ☐ Arcilla
2 ☐ Asfalto
3 ☐ Concreto
4 ☐ Tierra
5 ☐ Aserrín
6 ☐ Otro, ¿Cuál?______________________(5.3a)

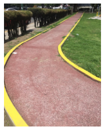
Arcilla

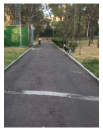
Asfalto

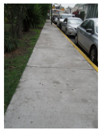
Concreto

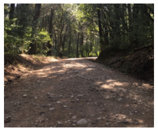
Tierra

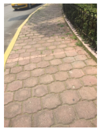
Adoquín

5.4. ¿El camino/ruta/pista de mayor jerarquía está obstruido?

1 ☐ Sí
2 ☐ No

5.5. ¿La condición del camino/ruta/pista de mayor jerarquía es buena?

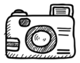

1 ☐ Totalmente de acuerdo
2 ☐ De acuerdo
3 ☐ Ni de acuerdo ni en desacuerdo
4 ☐ En desacuerdo
5 ☐ Totalmente en desacuerdo

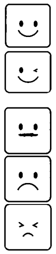

5.6. ¿Hay anuncios en el camino/ruta/pista de mayor jerarquía?

1 ☐ Sí
2 ☐ No ⟹ *Pasa a sección 6 "Seguridad e iluminación"*

5.7. ¿En general, los anuncios del camino/ruta/pista son visibles?

1 ☐ Sí
2 ☐ No

5.8. ¿En general, la condición de la mayoría de los anuncios es buena?

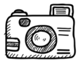

1 ☐ Totalmente de acuerdo
2 ☐ De acuerdo
3 ☐ Ni de acuerdo ni en desacuerdo
4 ☐ En desacuerdo
5 ☐ Totalmente en desacuerdo

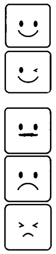

5.9. ¿El contenido de los anuncios es para indicar usos (por ejemplo: anuncios dentro de la pista que indican el sentido de los carriles para personas que caminan o corren, etc.)?

1 ☐ Sí
2 ☐ No

5.10. ¿El contenido de los anuncios es de comida/bebidas?

1 ☐ Sí
2 ☐ No

5.11. ¿El contenido de los anuncios es de avisos (por ejemplo: carreras próximamente, cierre de pista por mantenimiento)?

1 ☐ Sí
2 ☐ No

5.12. ¿El contenido de los anuncios es para distancias?

1 ☐ Sí
2 ☐ No

6. SEGURIDAD E ILUMINACIÓN

En esta sección se pretende conocer si el parque cuenta con la presencia de policías, así como diferentes elementos de seguridad.

6.1. ¿La seguridad dentro del parque es pública o privada?

1 ☐ Pública
2 ☐ Privada
3 ☐ Pública/privada
4 ☐ Ninguna

6.2. ¿Hay estaciones de policía dentro del parque?

1 ☐ Sí
2 ☐ No ⟹ *Pasa a 6.4*

6.3. ¿Cuántas estaciones de policías hay?

☐☐☐

6.4. ¿Existe presencia policial en el parque?

1 ☐ Sí, en todo momento
2 ☐ Solo en la noche
3 ☐ Ocasionalmente
4 ☐ No

6.5. Indique los elementos de seguridad presentes en el parque (si no hay elementos de seguridad pasa a la pregunta 6.7):

	Sí	No	
1	1 ☐	2 ☐	Cámaras de seguridad
2	1 ☐	2 ☐	Botones de pánico
3	1 ☐	2 ☐	Garitas de seguridad
4	1 ☐	2 ☐	Otros, ¿cuál? _________________ (6.5_4a)

6.6. ¿Existen carteles que indiquen la presencia de éstos elementos de seguridad? *(Al menos uno de los anteriores)*

1 ☐ Sí, ¿cuál? __________________________(6.6.a)
2 ☐ No

6.7. ¿Han habido incidentes de seguridad en lo que va del año? *(reportado en algún medio masivo de comunicación ó por parte de los administradores y/o grupos del parque)*

1 ☐ Sí
2 ☐ No

6a. Iluminación

Esta sección pretende identificar la iluminación del parque, así como el tipo, calidad y áreas iluminadas.

6a.1. ¿El parque cuenta con iluminación?

1 ☐ Todo el parque tiene iluminación
2 ☐ Solo algunos sectores tienen iluminación
3 ☐ No ⇨ *Pasa a sección 7 "Equipamientos y servicios internos"*
4 ☐ Otro, especicíficar ______________________________ (6a.1_4a)

6a.2. ¿Qué tipo de iluminación hay dentro del parque? *señale las que aplican.*

	Sí	No	
1	1 ☐	2 ☐	Panel Solar
2	1 ☐	2 ☐	Luz eléctrica
3	1 ☐	2 ☐	Planta de luz
4	1 ☐	2 ☐	Otra, ¿Cuál? __________________ (6a.2.4.1)

6a.3. Clasifica la iluminación del parque en general *(contestar solo si evalúas el parque durante la noche).*

1 ☐ Muy iluminado
2 ☐ Algo iluminado
3 ☐ Poco iluminado
9999 ☐ No aplica

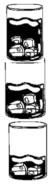

6a.4. ¿El área de estacionamientos (tanto de autos, motos y bicicletas) tiene iluminación?

1 ☐ Todo el área del estacionamiento tiene iluminación
2 ☐ Algunas secciones del estacionamiento tienen iluminación
3 ☐ No tiene iluminación
4 ☐ No cuenta con estacionamiento

7. EQUIPAMIENTOS Y SERVICIOS INTERNOS

7a. Baños

Esta sección pretende identificar las características de los baños, cantidad, costo y [illegible]

7a.1. ¿El parque cuenta con baños?

1 ☐ Sí
2 ☐ No ⟹ *Pasa a sección 7b*

7a.2. ¿Cuántos baños hay?

7a.3. ¿Cuántos de los baños están abiertos?

7a.4. ¿Los baños tienen un costo?

1 ☐ Sí
2 ☐ No ⟹ *Pasa a 7a.6*

7a.5. ¿Cuánto cuesta usar el baño?

$				

7a.6. ¿Hay baños para discapacitados?

1 ☐ Sí
2 ☐ No

7a.7. ¿Cuántos de los baños están etiquetados por género (hombre/mujer)?

1 ☐ Todos
2 ☐ Algunos
3 ☐ Ninguno

7a.8. ¿Cuántas de las tazas de baño funcionan?

1 ☐ Todas funcionan
2 ☐ Algunas tienen problemas o están en reparación
3 ☐ Ninguna funciona
4 ☐ No tiene tazas

7a.9. ¿Cuántos lavabos funcionan?

1 ☐ Todas funcionan
2 ☐ Algunas tienen problemas o están en reparación
3 ☐ Ninguna funciona
4 ☐ No tiene lavabo

7a.10. ¿La condición general de los baños es buena?

1 ☐ Totalmente de acuerdo
2 ☐ De acuerdo
3 ☐ Ni de acuerdo ni en desacuerdo
4 ☐ En desacuerdo
5 ☐ Totalmente en desacuerdo

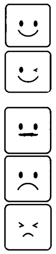

7a.11. Evaluar la cantidad de grafiti en los baños.

1 ☐ Mucho
2 ☐ Algo
3 ☐ Poco
4 ☐ Nada

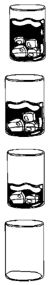

7a.12. Identifica todos los servicios presentes en los baños. *Selecciona todas las que apliquen.*

	Sí	No	
1	1 ☐	2 ☐	Jabón
2	1 ☐	2 ☐	Toallas de papel/Toallas de tela/Secadora de mano
3	1 ☐	2 ☐	Espejo
4	1 ☐	2 ☐	Papel higiénico
5	1 ☐	2 ☐	Botes de basura
6	1 ☐	2 ☐	Agua (para lava manos y jalar tazas)
7	1 ☐	2 ☐	Otra, ¿cuál? ______________________(7a12_7a)

7a.13. ¿Hay en al menos un baño cambiadores para bebes?

1 ☐ Sí
2 ☐ No

7b. Bebederos

Esta sección pretende identificar la existencia de bebederos, uso, condición e higiene.

7b.1. ¿El parque cuenta con bebederos?

1 ☐ Sí
2 ☐ No ⟹ *Pasa a sección 7c.*

7b.2. ¿Cuántos bebederos hay?

☐☐☐

7b.3. ¿Cuántos bebederos funcionan?

1 ☐ Todos funcionan
2 ☐ Algunos tienen problemas o están en reparación
3 ☐ Ninguno funciona
4 ☐ No se puede evaluar

7b.4. ¿La higiene de los bebederos es BUENA?

1 ☐ Totalmente de acuerdo
2 ☐ De acuerdo
3 ☐ Ni de acuerdo ni en desacuerdo
4 ☐ En desacuerdo
5 ☐ Totalmente en desacuerdo

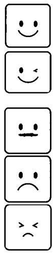

7b.5. ¿Qué tipo de bebedero hay?

	Sí	No	
1	1 ☐	2 ☐	Manual con llave
2	1 ☐	2 ☐	Manual con botón
3	1 ☐	2 ☐	Eléctrico
4	1 ☐	2 ☐	Garrafones
5	1 ☐	2 ☐	Otro, ¿Cuál? ____________________________(7b.5_5a)

7b.6. ¿Hay instrucciones de forma de uso del bebedero? 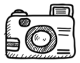

1 ☐ Sí
2 ☐ No
3 ☐ Sí, pero no son claras

7b.7. Observar la zona de bebederos por 5 minutos. Cuenta el número de personas que los utilizan, clasifícalas en 3 grupos (niños/adolescentes, adultos, adulto mayor)

	Número		
Niños/Adolescentes			
Adultos			
Adulto mayor			

7b.8. ¿Los bebederos están patrocinados por alguna marca?

1 ☐ Sí, ¿cuál? ______________________________(7b.8a)
2 ☐ No

7c. Basurero

Esta sección pretende identificar la cantidad y tipo de basura en el

7c.1. ¿El lugar cuenta con botes de basura?

1 ☐ Sí
2 ☐ No ⟹ *Pasa a sección 7d.*

7c.2. ¿Aproximadamente, cuántos botes de basura hay en todo el parque?

7c.3. ¿Cuántos botes separan la basura? (orgánica e inorgánica u otro tipo de separación como PET, latas, etc.)

1 ☐ Todos están separados
2 ☐ Algunos están separados
3 ☐ Ninguno está separado

7c.4. ¿En general, cuánta basura hay dentro de los botes?

1 ☐ Mucho 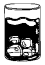
2 ☐ Algo 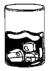
3 ☐ Poco 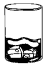
4 ☐ Nada 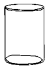

7c.5. ¿Hay basura en el parque, que no sea alrededor y/o dentro de los botes de basura?

1 ☐ Si, ¿Dónde? ____________________________________(7c.5a)
2 ☐ No

7c.6. ¿Cuánta basura “de riesgo” es visible en el parque? Por ejemplo: recipientes de alcohol, preservativos, medicamentos, vidrios, etc.)

1 ☐ Mucho 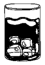
2 ☐ Algo 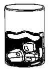
3 ☐ Poco 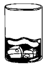
4 ☐ Nada 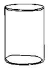

7c.7. ¿Hay botes de basura para productos reciclables (ejemplo: periódico, vidrio, etc.)?

1 ☐ Sí, ¿qué tipo de productos? ____________________ (7c.7a)
2 ☐ No

7d. Refugios de comida

Está sección pretende identificar el uso de refugios y su condición.

*Nota al encuestador:
Refugio de comida se refiere al lugar donde realizan convivios o fiestas.

7d.1. ¿El parque tiene refugios?

1 ☐ Si
2 ☐ No ⟹ *Pasa a sección 7e.*

7d.2. ¿Qué se localiza debajo de los refugios? *Señala todas las que aplican.*

	Sí	No	
1	1 ☐	2 ☐	Asadores
2	1 ☐	2 ☐	Bancas
3	1 ☐	2 ☐	Mesas
4	1 ☐	2 ☐	Baños
5	1 ☐	2 ☐	Fuentes de bebidas
6	1 ☐	2 ☐	Otro, ¿Cuál? _________________ (7d.2_6a)

7d.3. ¿La condición en general de los refugios es BUENA?

1 ☐ Totalmente de acuerdo
2 ☐ De acuerdo
3 ☐ Ni de acuerdo ni en desacuerdo
4 ☐ En desacuerdo
5 ☐ Totalmente en desacuerdo

7d.4. ¿Existen señales que indiquen una política de reservación?

1 ☐ No
2 ☐ Sí, indican que las reservaciones son requeridas
3 ☐ Sí, indican que las reservaciones no son requeridas

7e. Bancas

Esta sección pretende identificar el número y condición de bancas dentro del parque.

7e.1. ¿Hay bancas en el parque?

1 ☐ Sí
2 ☐ No ⟹ *Pasa a sección 7f.*

7e.2. ¿La condición de los lugares para sentarse es buena?

1 ☐ Totalmente de acuerdo
2 ☐ De acuerdo
3 ☐ Ni de acuerdo ni en desacuerdo
4 ☐ En desacuerdo
5 ☐ Totalmente en desacuerdo

7f. Gimnasio

Está sección pretende conocer si los parques cuentan con gimnasio al aire libre o cerrados.

7f.1. ¿El parque cuenta con gimnasio al aire libre?

1 ☐ Si
2 ☐ No ⟹ *Pasa a 7f.5*

7f.2. ¿El gimnasio al aire libre se encuentra en buena condición?

1 ☐ Totalmente de acuerdo
2 ☐ De acuerdo
3 ☐ Ni de acuerdo ni en desacuerdo
4 ☐ En desacuerdo
5 ☐ Totalmente en desacuerdo

7f.3. ¿El gimnasio al aire libre está siendo utilizado?

1 ☐ Si
2 ☐ No

7f.4. ¿Qué tipo de actividad se puede realizar en el gimnasio al aire libre? (señala todas las que apliquen)

	Sí	No	
1	1 ☐	2 ☐	Caminadora flotante
2	1 ☐	2 ☐	Caminadora elíptica
3	1 ☐	2 ☐	Potro
4	1 ☐	2 ☐	Remadora
5	1 ☐	2 ☐	Tablas abdominales
6	1 ☐	2 ☐	Multifuncional sencillo
7	1 ☐	2 ☐	Bicicleta
8	1 ☐	2 ☐	Esquiadora
9	1 ☐	2 ☐	Press de espalda
10	1 ☐	2 ☐	Barras paralelas
11	1 ☐	2 ☐	Press de pecho
12	1 ☐	2 ☐	Aros ejercicio
13	1 ☐	2 ☐	Press de pierna
14	1 ☐	2 ☐	Aros y cintura
15	1 ☐	2 ☐	Twist de cintura
16	1 ☐	2 ☐	Masaje de espalda
17	1 ☐	2 ☐	Masaje de hombro
18	1 ☐	2 ☐	Tabla de surf
19	1 ☐	2 ☐	Otro, especificar ________________________(7f.4_19.1)
19a	1 ☐	2 ☐	Otro, especificar ________________________(7f.4_19.1a)
19b	1 ☐	2 ☐	Otro, especificar ________________________(7f.4_19.1b)

7f.5. ¿El parque cuenta con gimnasio cerrado?

1 ☐ Si
2 ☐ No ⟹ *Pasa a sección 8 "Salud, nutrición e higiene".*

7f.6. ¿Qué tipo de actividades se pueden realizar en el gimnasio cerrado? *Enlista todas las actividades.*

____
____
____
____

7f.7. ¿El parque cuenta con entrenadores o preparadores físicos?

1 ☐ Sí, ¿dónde se encuentran? ________________________(7f.7.1)
2 ☐ No

8. SALUD, NUTRICIÓN E HIGIENE.

8a. Salud.

8a.1 ¿Existe servicio médico dentro del parque?

1 ☐ Sí
2 ☐ No

8b. Nutrición: publicidad

Está sección pretende identificar la publicidad dentro y fuera de los parques, así como los establecimientos de alimentos.

8b.1. ¿Existe publicidad de alimentos y bebidas dentro del parque?

1 ☐ Si
2 ☐ No ⟹ *Pasa a 8b.4*

8b.2. Señale todos los elementos donde haya publicidad dentro del parque.

	Sí	No	
1	1 ☐	2 ☐	En el camino/ruta/pista
2	1 ☐	2 ☐	En los botes de basura
3	1 ☐	2 ☐	En tableros específicos para publicidad
4	1 ☐	2 ☐	En la cara interior de las bardas
5	1 ☐	2 ☐	En las bancas
6	1 ☐	2 ☐	En kioscos centrales
7	1 ☐	2 ☐	En la entrada del parque
8	1 ☐	2 ☐	Otro, ¿Cuál? ______________________(8b.2_8a)

8b.3. Para cada anuncio observado, rellena los siguientes incisos (2-4) usando la escala que aparece hasta abajo del cuadro.

1. Número de anuncio	2. Tipo de producto del anuncio	3. Canal de comunicación	4. Tipo de promoción	5. Tipo de personaje
Números (1 al 100)	1- chocolates, dulces, confiterías, barras de energía y untables<br>2- pasteles, galletas dulces, mezclas y masas para hacer pasteles<br>3- botanas saladas (palomitas, frituras de maíz, nueces, cacahuates y papas fritas)<br>4- jugos procesados<br>5- lácteos con azúcar (lácteos fermentados, yogurt con azúcares, leches saborizadas y quesos)<br>6- lácteos y derivados (leches, quesos y cremas)<br>7- bebidas energéticas<br>8- refrescos y otras bebidas gaseosas (incluyendo bebidas light<br>9- cereales de desayuno<br>10- comida preparada (hamburguesas, pastas, pizzas y sándwiches)<br>11- aceites y mantequillas<br>12- panes y productos similares	1- cartel/poster<br>2- pintadas en la pared<br>3- mantas/pancarta<br>4- refrigeradores<br>5- anuncio iluminado<br>6- persona disfrazada<br>7- carpa con persona animando y haciendo concurso<br>8- anuncio con movimiento (pantalla)<br>9- barda<br>10- otro: especificar	1- sin promoción<br>2- concurso<br>3- sorteos<br>4- regalos<br>5- precio (descuento)<br>6- mas producto (2x1)<br>7- ligada con terceros (en la compra de uno llevas otro de regalo)<br>8- cupones<br>9- registro por internet<br>10- personaje (deportistas famosos, caricaturas o artistas)<br>11- invitación a eventos o conciertos<br>12- obsequios de productos nuevos<br>13- otro: especificar	1- caricaturas<br>2- personajes con una licencia (dora la exploradora)<br>3- deportistas famosos<br>4- celebridades no deportivas<br>5- personales de películas<br>6- equipo de famosos

	13- pastas, arroz y granos<br>14- carne, pollo, pescado procesado (salchicha, jamón, atún enlatado y otros embutidos)<br>15- frutas y verduras procesadas<br>16- salsa, dip y aderezo<br>17- bebidas alcohólicas<br>18- agua natural<br>19- otro: especificar			7- ni deportistas ni históricos (personajes de navidad)<br>8- imágenes de niños<br>9- otro: especificar

8b.4. ¿Existe publicidad de alimentos y bebidas alrededor del parque?

1 ☐ Si
2 ☐ No ⟹ *Pasa a sección 8c.*

8b.5. Señale todos los elementos donde haya publicidad alrededor del parque.

	Sí	No	
1	1 ☐	2 ☐	Alrededor de las bardas del parque
2	1 ☐	2 ☐	Mupis (publicidad en las paradas de camión)
3	1 ☐	2 ☐	Posters
4	1 ☐	2 ☐	Vallas/rejas
5	1 ☐	2 ☐	En las bancas
6	1 ☐	2 ☐	Otro, ¿Cuál? ______________________(8b.5_6a)

8b.6. Para cada anuncio observado, rellena los siguientes incisos (2-4) usando la escala que aparece hasta abajo del cuadro.

1. Número de anuncio	2. Tipo de producto del anuncio	3. Canal de comunicación	4. Tipo de promoción	5. Tipo de personaje
Números (1 al 100)	1- chocolates, dulces, confiterías, barras de energía y untables	1- cartel/poster<br>2- pintadas en la pared	1- sin promoción<br>2- concurso<br>3- sorteos	1- caricaturas

	2- pasteles, galletas dulces, mezclas y masas para hacer pasteles<br>3- botanas saladas (palomitas, frituras de maíz, nueces, cacahuates y papas fritas)<br>4- jugos procesados<br>5- lácteos con azúcar (lácteos fermentados, yogurt con azúcares, leches saborizadas y quesos)<br>6- lácteos y derivados (leches, quesos y cremas)<br>7- bebidas energéticas<br>8- refrescos y otras bebidas gaseosas (incluyendo bebidas light<br>9- cereales de desayuno<br>10- comida preparada (hamburguesas, pastas, pizzas y sándwiches)<br>11- aceites y mantequillas<br>12- panes y productos similares<br>13- pastas, arroz y granos<br>14- carne, pollo, pescado procesado (salchicha, jamón, atún enlatado y otros embutidos)<br>15- frutas y verduras procesadas<br>16- salsa, dip y aderezo<br>17- bebidas alcohólicas<br>18- agua natural<br>19- otro: especificar	3- mantas/pancarta<br>4- refrigeradores<br>5- anuncio iluminado<br>6- persona disfrazada<br>7- carpa con persona animando y haciendo concurso<br>8- anuncio con movimiento (pantalla)<br>9- barda<br>10- otro: especificar	4- regalos<br>5- precio (descuento)<br>6- mas producto (2x1)<br>7- ligada con terceros (en la compra de uno llevas otro de regalo)<br>8- cupones<br>9- registro por internet<br>10- personaje (deportistas famosos, caricaturas o artistas)<br>11- invitación a eventos o conciertos<br>12- obsequios de productos nuevos<br>13- otro: especificar	2- personajes con una licencia (dora la exploradora)<br>3- deportistas famosos<br>4- celebridades no deportivas<br>5- personales de películas<br>6- equipo de famosos<br>7- ni deportistas ni históricos (personajes de navidad)<br>8- imágenes de niños<br>9- otro: especificar

8c. Nutrición: Establecimientos alimenticios

8c.1. ¿Hay venta de alimentos y/o bebidas dentro del parque?

1 ☐ Si
2 ☐ No ⟹ *Pasa a sección 8c.3*

8c.2. Para cada establecimiento, rellena los siguientes incisos (2-3) usando la escala que aparece hasta abajo del cuadro.

1. Número de puesto	2. Tipo de puesto (fijo o ambulante):	3. Tipo de productos que se venden en el puesto (seleccionar solo 1)

Números (1 al 100)	1 tienda de abarrotes<br>2 supermercado<br>3 minisuper<br>4 tienda de conveniencia (oxxo, seven)<br>5 puesto fijo (metal)<br>6 puesto desmontable<br>7 carrito<br>8 bicicleta<br>9 carretilla<br>10 en el piso<br>11 otro, especificar:	1 fritanga<br>2 hamburguesa y hot dog<br>3 puesto de chicharrones y papas<br>4 frutas y verduras<br>5 taquería<br>6 esquites<br>7 dulces empaquetados<br>8 gorditas de nata<br>9 helados, nieves y paletas<br>10 jugos y licuados<br>11 tamal y atole<br>12 bebidas azucaradas, energética y agua<br>13 tortas y sándwich<br>14 palomitas<br>15 puesto de algodón<br>16 nueces, semillas y dulces<br>17 otro, especificar:

8c.3. ¿Hay venta de alimentos y/o bebidas alrededor del parque?

1 ☐ Si

2 ☐ No ⟹ *Pasa a sección 8d.*

8c.4. Para cada establecimiento, rellena los siguientes incisos (2-3) usando la escala que aparece hasta abajo del cuadro.

1. Número de puesto	2. Tipo de puesto (fijo o ambulante):	3. Tipo de productos que se venden en el puesto (seleccionar solo 1)
Números (1 al 100)	1 tienda de abarrotes<br>2 supermercado<br>3 minisuper<br>4 tienda de conveniencia (oxxo, seven)<br>5 puesto fijo (metal)<br>6 puesto desmontable<br>7 carrito<br>8 bicicleta<br>9 carretilla<br>10 en el piso	1 fritanga<br>2 hamburguesa y hot dog<br>3 puesto de chicharrones y papas<br>4 frutas y verduras<br>5 taquería<br>6 esquites<br>7 dulces empaquetados<br>8 gorditas de nata<br>9 helados, nieves y paletas<br>10 jugos y licuados

	11 Restaurant<br>12 Cafetería<br>13 Fonda<br>14 Paletería<br>15 Tamalería<br>16 Pastelería<br>17 otro, especificar:	11 tamal y atole<br>12 bebidas azucaradas, energética y agua<br>13 tortas y sándwich<br>14 palomitas<br>15 puesto de algodón<br>16 nueces, semillas y dulces<br>17Marisquería<br>18 Pizza<br>19 Tortas<br>20 Mixiotes<br>21 Sopes<br>22 Misceláneos<br>23 otro, especificar:

8d. Higiene: Mascotas

Está sección pretende identificar si hay presencia de mascotas en el parque, así como los comportamientos de los usuarios.

8d.1. ¿Hay anuncio que restringe el acceso a mascotas? 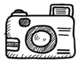

1 ☐ Sí
2 ☐ No

8d.2. ¿Hay algún anuncio especificando si los perros están obligados a estar con correa o a permanecer en un área específica del parque? 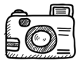

1 ☐ Sí
2 ☐ No
3 ☐ No sé (porque los anuncios no especifican o no se ven claramente)

8d.3. ¿Hay algún anuncio especificando donde desechar el excremento de las mascotas?

1 ☐ Sí
2 ☐ No
3 ☐ No sé (porque los anuncios no especifican o no se ven claramente)

8d.4. ¿Existen lugares específicos para tirar el excremento?

1 ☐ Sí, ¿Cuál? ____________________(8d.4a)
2 ☐ No

8d.5. ¿Existe un lugar especial para perros?

1 ☐ Si, ¿Cuál? ______________________(8d.5a)
2 ☐ No

8e. Higiene: comportamientos permitidos o restringidos.

Está sección pretende identificar los comportamientos dentro del parque como: escupir, arrojar chicles y fumar.

8e.1. ¿Hay señalización para no escupir?

1 ☐ Sí
2 ☐ No

8e.2. Observa por 5 minutos y cuenta el número de personas que escupen, clasifícalas en 3 grupos (niños/adolescentes, adultos, adulto mayor)

	Número
1. Niños/Adolescentes	
2. Adultos	
3. Adulto mayor	
4. No se observó gente escupiendo	

8e.3. ¿Hay señalización para no arrojar chicles en el parque?

1 ☐ Si
2 ☐ No

8e.4. Observar por 5 minutos y cuenta el número de personas que arroja chicles al parque, si observas personas clasifícalas en 3 grupos (niños/adolescentes, adultos, adulto mayor)

	Número
1. Niños/Adolescentes	
2. Adultos	
3. Adulto mayor	
4. No se observó gente arrojando chicles	

8e.5. ¿Hay señalización restrictiva para no fumar?

1 ☐ Si
2 ☐ No

8e.6. Observar por 5 minutos y cuenta el número de personas que fuman, si observas personas clasifícalas en 3 grupos (adolescentes, adultos, adulto mayor)

	Número
1. Niños/Adolescentes	
2. Adultos	
3. Adulto mayor	
4. No se observó gente fumando	

8e.7. ¿Existen áreas donde esté permitido fumar?

1 ☐ Si, ¿Dónde? ___________________________(8e.7a)
2 ☐ No

9. ADMINISTRACIÓN Y MANTENIMIENTO

Esta sección pretende conocer quien administra el parque, así como las distintas funciones de administración y mantenimiento.

9.1. ¿Quién administra el lugar?

	Sí	No	
1	1 ☐	2 ☐	SEMARNAT (Secretaría de Medio Ambiente y Recursos Naturales)
2	1 ☐	2 ☐	SEDEMA (Secretaría de Medio Ambienta de la Ciudad de México)
3	1 ☐	2 ☐	Alcaldía
4	1 ☐	2 ☐	Se desconoce
5	1 ☐	2 ☐	Otro, ¿Cuál? ______________________(9.1_5a)

9.2. Preguntar a los administradores ¿De dónde vienen los recursos para el mantenimiento del parque?
____
____
____
____

9.3. ¿Observas trabajadores del parque uniformados?

1 ☐ Sí, ¿qué dice su uniforme? ______________________________________(9.3a)
2 ☐ No

9.4. ¿Qué tareas desempeñaban los trabajadores presenten en el parque?

1. Tipo de trabajador	2. Zona	3. Número de trabajadores	4. Horarios

9.5. ¿Existe algún tipo de señalamiento de apropiación del parque?

1 ☐ Sí, ¿cuál? (por ejemplo: anuncios de la CDMX, SEDEMA, etc.) ___________________________________(9.5.1)
2 ☐ No

FIN DEL CUESTIONARIO
