# Supplementary material for: Development and Validation of the Mexican Public Open Spaces Tool (MexPOS)
Source: Int J Environ Res Public Health. 2022 Jul 5;19(13):8198. doi: 10.3390/ijerph19138198 (PMC9266626; doi:10.3390/ijerph19138198)
Supplement: Supplementary file 1 [file ijerph-19-08198-s001.zip › File S2. Final questionnaire-English.pdf]

Instituto Nacional de Salud Pública
Centro de Investigación en Nutrición y Salud
Línea de Investigación de medio ambiente, actividad física, nutrición y salud.

EVALUATION QUESTIONNAIRE, THROUGH DIRECT OBSERVATION OF THE ENVIRONMENTAL CHARACTERISTICS OF THE PARK.

**Check List:**	SI	NO
BEFORE ARRIVING AT THE PARK….		
Did you read the manual?		
Did you fill out the general information card? (Section 1)		
BEFORE FILLING OUT THE QUESTIONNAIRE		
Did you walk around the park before answering this questionnaire?		
BEFORE THE QUESTIONNAIRE CAPTURE IS COMPLETED…		
Did you answer all sections of the questionnaire?		
Did you review all your answers?		
In case of any mistake in a question, did you correct it?		

¿What to do in case of a mistake an answer?
Write an "X" at the right answer, in case of error, fill the square and put an "X" at the right answer.

[X] → ■ **Error**   [X] **Correction**

Pollster: [| |] [| |]   Park Code: [| |]

1. Date of observation [|] / [|] / [|]

2. Temperature [|] °C (Enter to :https://weather.com/esUS/tiempo/hoy/l/Ciudad+de+Mexico+Mxico+MXDF0132:1:MX)

3. Weather
   1 [ ] Sunny
   2 [ ] Partly sunny
   3 [ ] Partly cloudy
   4 [ ] Cloudy
   5 [ ] Rainy

(Enter to: https://weather.com/esUS/tiempo/hoy/l/Ciudad+de+Mexico+Mxico+MXDF0132:1:MX)

* *Reverse item*

4. Air quality

				IMECA

(enter to: http://aqicn.org/map/world/es/#@g/12.0511/-82.1997/5z)

5. Initial capture schedule :

		:			Am	Pm

6. End capture schedule:

		:			Am	Pm

7. Period Captured of the day:

	1.Day			2.Night

1. General Park Information

Before arriving at the park fill this section

1.1 Name of the park: ____

1.2 Location: ____

1.3 Typology:

1		Metropolitan Park
2		Local Park
3		Neighborhood Park
4		Pocket Park
5		Remaining Park
6		Roundabout
7		Garden
8		Square

* *Reverse item*

9 [ ] Boulevard

2. Food Environment and Health wellness

2.1 Is the land use around the park mainly commercial?*

1 [ ] Yes
2 [ ] No

2.2 Is the land use around the park mainly institutional? (e. g schools and government offices)

1 [ ] Yes
2 [ ] No

2.3 Is there food and beverage advertising inside the park?*

1 [ ] Yes
2 [ ] No

2.4 Are there advertisements promoting cakes, cookies or cake dough inside the park?

1 [ ] Yes
2 [ ] No

2.5 Are there food and beverage stalls around the park? *

1 [ ] Yes
2 [ ] No

2.6 Write down the number of stalls around the park.*

1 to 100 number ______________________________________

* *Reverse item*

3. MAINTENANCE

3.1 Is the condition of the road, route or major trail good?

1 ☐ Totally Agree
2 ☐ Agree
3 ☐ Neither agree or disagree
4 ☐ Disagree
5 ☐ Totally disagree

3.2 Are the walking paths in good condition?

1 ☐ Totally Agree
2 ☐ Agree
3 ☐ Neither agree or disagree
4 ☐ Disagree
5 ☐ Totally disagree

3.3 Are the eating areas in good condition?

1 ☐ Totally Agree
2 ☐ Agree
3 ☐ Neither agree or disagree
4 ☐ Disagree
5 ☐ Totally disagree

* *Reverse item*

3.4 Are the seating areas in good condition?

1 Totally Agree
2 Agree
3 Neither agree or disagree
4 Disagree
5 Totally disagree

3.5 Is the state of the places to sit good?

1 Totally Agree
2 Agree
3 Neither agree or disagree
4 Disagree
5 Totally disagree

3.6 Are the green areas in good condition?

1 Totally Agree
2 Agree
3 Neither agree or disagree
4 Disagree
5 Totally disagree

3.7 Is the playground area in good condition?

1 Totally Agree
2 Agree

* *Reverse item*

3	☐	Neither agree or disagree	😐
4	☐	Disagree	☹
5	☐	Totally disagree	☹

3.8 Is the outdoor gymnasium in good condition?

1	☐	Totally Agree	☺
2	☐	Agree	☺
3	☐	Neither agree or disagree	😐
4	☐	Disagree	☹
5	☐	Totally disagree	☹

3.9 In general, is the park well maintained?

1	☐	Yes
2	☐	No

3.10 Are there properties around the park in poor condition?

1	☐	Yes
2	☐	No

3.11 Is the condition of the landscape adequate?

1	☐	Totally Agree	☺
2	☐	Agree	☺
3	☐	Neither agree nor disagree	😐
4	☐	Disagree	☹
5	☐	Totally disagree	☹

3.12 Does the opening or operation of the park depend on a janitor or manager?

* *Reverse item*

1 ☐ Yes
2 ☐ No

3.13 Is the condition of the sidewalks good?

1 ☐ Yes
2 ☐ No

3.14 Is the condition of the sidewalks regular?

1 ☐ Yes
2 ☐ No

3.15 Is the condition of the sidewalks bad?*

1 ☐ Yes
2 ☐ No

3.16 Is there garbage around the park? *

1 ☐ Yes
2 ☐ No

3.17 Is there garbage in the park, other than around and/o inside the trash cans?*

1 ☐ Yes, where? ______________________________
2 ☐ No

* *Reverse item*

3.18 How much hazardous waste is visible in the park? (e.g. alcohol containers, preservatives, medicines, glass, etc)*

1	☐	A lot
2	☐	Some
3	☐	A little
4	☐	Nothing

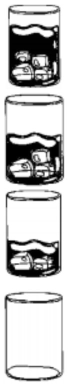

3.19 Do you observe animal excrement in the park? *

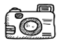

1	☐	Yes, where? ____________________
2	☐	No
3	☐	Not applicable

3.20 Are there empty or unpleasant buildings around the park?*

1	☐	Yes
2	☐	No

3.21 Do you see graffiti around the park?*

1	☐	Yes

* *Reverse item*

2 ☐ No

4. AMENITIES

4.1 Is there a fronton court inside the park?

1 ☐ Yes
2 ☐ No

4.2 Is there a football/soccer court inside the park?

1 ☐ Yes
2 ☐ No

4.3 Is there a 5-a-side football/soccer court inside the park?

1 ☐ Yes
2 ☐ No

4.4 Is there a jogging path inside the park?

1 ☐ Yes
2 ☐ No

4.5 Is there a bike path inside the park?

1 ☐ Yes
2 ☐ No

* *Reverse item*

4.6 Is there a skatepark area inside the park?

1 ☐ Yes
2 ☐ No

4.7 Are the other areas of activities not mentioned above?

1 ☐ Yes
2 ☐ No

4.8 What other areas for activities did you observe?

Mention ____

4.9 Is the outdoor gym being used?

1 ☐ Yes
2 ☐ No

4.10 Are there abdominal boards in the outdoor gym?

1 ☐ Yes
2 ☐ No

4.11 Is there a shoulder massage machine in the outdoor gym?

1 ☐ Yes
2 ☐ No

* *Reverse item*

4.12 Is there a stationary bike in the outdoor gym?

1 ☐ Yes
2 ☐ No

4.13 Is there a rowing machine in the outdoor gym?

1 ☐ Yes
2 ☐ No

4.14 Is there a ski walker in the outdoor gym?

1 ☐ Yes
2 ☐ No

4.15 Is there a colt in the outdoor gym?

1 ☐ Yes
2 ☐ No

4.16 Is there an elliptical trainer in the outdoor gym?

1 ☐ Yes
2 ☐ No

4.17 Is there a leg press machine in the outdoor gym?

1 ☐ Yes
2 ☐ No

4.18 Does the outdoor gym have hoops?

1 ☐ Yes

* *Reverse item*

2 ☐ No

4.19 Is there a chest press machine in the outdoor gym?

1 ☐ Yes
2 ☐ No

4.20 Does the outdoor gym have a multifunctional machine?

1 ☐ Yes
2 ☐ No

4.21 Does the outdoor gym have hoops and waistband?

1 ☐ Yes
2 ☐ No

4.22 Are there bars in the outdoor gym?

1 ☐ Yes
2 ☐ No

4.23 Is there a floating treadmill in the outdoor gym?

1 ☐ Yes
2 ☐ No

4.24 Is there a 7 football/soccer field in the park?

1 ☐ Yes
2 ☐ No

* *Reverse item*

4.25 Are the courts that combine soccer and basketball?

1 ☐ Yes
2 ☐ No

4.26 Is there a basketball court inside the park?

1 ☐ Yes
2 ☐ No

4.27 Does the outdoor gym have a surfboard?

1 ☐ Yes
2 ☐ No

4.28 Does the outdoor gym have a back massage machine?

1 ☐ Yes
2 ☐ No

4.29 Is the waist twist inside the outdoor gym?

1 ☐ Yes
2 ☐ No

4.30 Is there any other apparatus not mentioned above inside the outdoor gym?

1 ☐ Yes, which one? _____________
2 ☐ No

* *Reverse item*

4.31 Does the park have lighting?

1 ☐ The whole park is illuminated
2 ☐ Only some parts are illuminated
3 ☐ No
4 ☐ Other, please specify______________________________________

4.32 Is the solar panel inside the park?

1 ☐ Yes
2 ☐ No

4.33 Is the electric lighting inside the park?

1 ☐ Yes
2 ☐ No

4.34 Are all areas of the park open to the public?

1 ☐ Yes
2 ☐ No

4.35 Are there botanical gardens inside the park?

1 ☐ Yes
2 ☐ No

4.36 Does the park have shelters?

1 ☐ Yes
2 ☐ No

* *Reverse item*

4.37 Does the park have bathrooms?

1 ☐ Yes
2 ☐ No

4.38 Does the park have drinking fountains?

1 ☐ Yes
2 ☐ No

4.39 Are there any creeks, lakes or ponds inside the park?

1 ☐ Yes. where? _______________
2 ☐ No

4.40 Is the park fenced or gated?

1 ☐ Yes, percentage____________
2 ☐ No

4.41 Is the bicycle parking around the park?

1 ☐ Yes
2 ☐ No

4.42 Is there a bike route that leads to the park?

1 ☐ Yes, which one? __________________
2 ☐ No

4.43 Does the park have a parking lot?

1 ☐ Yes
2 ☐ No

* *Reverse item*

4.44 Does the park have a parking lot for motorcycles?

1 ☐ Yes
2 ☐ No

5. SIGNALING

5.1 Is there a map of the park?

1 ☐ Yes
2 ☐ No

5.2 Is there any reference or sign indicating the name of the park?

1 ☐ Yes, which one? (e.g. formal or informal poster)
2 ☐ No

5.3 Is there any sign of the appropriation of the park?

1 ☐ Yes, which one? (e.g. announcement of CDMX, SEDENA, etc)___________________________
2 ☐ No

5.4 Are there any indications of who oversees the park?

1 ☐ Yes
2 ☐ No

5.5 Are there signs indicating the park´s schedule of operation?

* *Reverse item*

1 ☐ Yes
2 ☐ No

5.6 Does the park have historical, educational, or artistic features? (e.g. monuments, statues, cultures, nature, fountains)

1 ☐ Yes, please specify ___________________________________
2 ☐ No

5.7 Are there posters promoting events inside the park?

1 ☐ Yes, What kind of events? ___________________
2 ☐ No

5.8 Are there any announcements indicating the road, route, track of higher hierarchy??

1 ☐ Yes
2 ☐ No

5.9 Are there signs indicating whether the park has equipment for rental? (e.g. boats, bicycles, carts)

1 ☐ Yes, please specify which one ___________________
2 ☐ No

5.10 Are there garbage containers for recyclable products? (e.g. paper, newspaper, glass, etc)

1 ☐ Yes, what kind of product? ____
2 ☐ No

5.11 Are there signs indicating whether dogs must be on a leash or in a specific area?

* *Reverse item*

1 ☐ Yes
2 ☐ No
3 ☐ I don´t know (the advertisements are not legible or understandable)

5.12 Are there signs indicating where to dispose of pet excrement??

1 ☐ Yes
2 ☐ No
3 ☐ I don´t know (the advertisements are not legible or understandable)

5.13 Are there signs restricting bicycle parking area inside the park??

1 ☐ Yes
2 ☐ No

5.14 Are there No parking signs around the park?

1 ☐ Yes
2 ☐ No

5.15 Are there signs indicating other signage not mentioned above?

Mention____

6. SAFETY

6.1 Is there any medical service inside the park??

1 ☐ Yes
2 ☐ No

* *Reverse item*

6.2 Do you observe uniformed park workers?

1 ☐ Yes, what does your uniform say?? ____
2 ☐ No

6.3 Are there police stations inside the park?

1 ☐ Yes
2 ☐ No

6.4 Is there a police presence inside the park??

1 ☐ Yes, at all times
2 ☐ Only at night
3 ☐ Occasionally
4 ☐ No

6.5 Are there security cameras inside the park?

1 ☐ Yes
2 ☐ No

6.6 Do you see panic buttons in the park?

1 ☐ Yes
2 ☐ No

6.7 Are there guardhouses in the park??

1 ☐ Yes
2 ☐ No

6.8 Is there any evidence of threatening people or behavior? (e.g. gangs, alcohol, drug use).

* *Reverse item*

1 ☐ Yes, please specify_ ______________________________
2 ☐ No

6.9 Is there vandalism inside the park? (e.g. damage signs)

1 ☐ Yes
2 ☐ No

6.10 Is there poor street lighting in the area around the park?

1 ☐ Yes
2 ☐ No

6.11 Have there been any security incidents so far this year? (reported in the media or by the park administrators?

1 ☐ Yes
2 ☐ No

7. PERCEIVED ENVIROMET

7.1 Based on what you observed today, is the park attractive to you?

1 ☐ Totally Agree
2 ☐ Agree
3 ☐ Neither agree or disagree
4 ☐ Disagree
5 ☐ Totally disagree

* *Reverse item*

7.2 Are the trees the main source of shade in the park?

1	☐	Totally Agree
2	☐	Agree
3	☐	Neither agree or disagree
4	☐	Disagree
5	☐	Totally disagree

7.3 In total, how much of the park could be shaded??

0	☐	0
1	☐	1-25%
2	☐	26-75%
3	☐	76-100%

7.4 In general, what is the percentage of wooded areas in the park? (e.g. forest or dense trees)

0	☐	0
1	☐	1-25%
2	☐	26-75%
3	☐	76-100%

7.5 Do you hear music sounds inside the park?

1	☐	Yes
2	☐	No

* *Reverse item*

Instituto Nacional de Salud Pública
Centro de Investigación en Nutrición y Salud
Línea de Investigación de medio ambiente, actividad física, nutrición y salud.

7.6 Do you hear voices sounds inside the park?

1 ☐ Yes
2 ☐ No

7.7 Do you hear bird sounds inside the park?

1 ☐ Yes
2 ☐ No

7.8 Do you hear water sounds inside the park?

1 ☐ Yes
2 ☐ No

7.9 Do you hear traffic sounds inside the park? *

1 ☐ Yes
2 ☐ No

7.10 Do you hear other sounds not mentioned above?

1 ☐ Yes, which one? ________________________
2 ☐ No

7.11 Is there excessive noise around the park?*

1 ☐ Yes
2 ☐ No

7.12 Is heavy traffic around the park?*

1 ☐ Yes

* *Reverse item*

2 ☐ No

7.13 Are people smoking inside the park?*

1 ☐ Totally Agree
2 ☐ Agree
3 ☐ Neither agree or disagree
4 ☐ Disagree
5 ☐ Totally disagree

7.14 According to today's experience, is the smell of the park pleasant?

1 ☐ Totally Agree
2 ☐ Agree
3 ☐ Neither agree or disagree
4 ☐ Disagree
5 ☐ Totally disagree

8. URBAN SURROUNDINGS

8.1 Does the park have a street inside?

1 ☐ Yes
2 ☐ No

8.2 Is there a shared trail around the park?

1 ☐ Yes
2 ☐ No

* *Reverse item*

8.3 Does the park have handrails?

1 ☐ Yes
2 ☐ No

8.4 Does the park have ramps to guarantee access to the park?

1 ☐ Yes
2 ☐ No

8.5 Is the main land use inside the park natural?

1 ☐ Yes
2 ☐ No

8.6 Are most of the streets around the park primary?*

1 ☐ Totally Agree
2 ☐ Agree
3 ☐ Neither agree or disagree
4 ☐ Disagree
5 ☐ Totally disagree

8.7 Are most of the streets around the park secondary?

* *Reverse item*

1 ☐ Totally Agree
2 ☐ Agree
3 ☐ Neither agree or disagree
4 ☐ Disagree
5 ☐ Totally disagree

8.8 Are most of the streets around the park tertiary?

1 ☐ Totally Agree
2 ☐ Agree
3 ☐ Neither agree or disagree
4 ☐ Disagree
5 ☐ Totally disagree

8.9 Are there any streets with pedestrian traffic lights around the park?

1 ☐ Yes
2 ☐ No

8.10 Are there streets with traffic lights around the park?

1 ☐ Yes
2 ☐ No

8.11 Are there streets with safe crossings around the park?

1 ☐ Yes
2 ☐ No

* *Reverse item*

8.12 Are there streets with zebra crossings around the park?

1 ☐ Yes
2 ☐ No

8.13 Are there pedestrian streets around the park?

1 ☐ Yes
2 ☐ No

8.14 Are there streets with sidewalks around the park?

1 ☐ Yes
2 ☐ No ➡ skip the next question

8.15 Is the condition of these sidewalks regular? *

1 ☐ Yes
2 ☐ No

8.16 Are there public transport stations within a 100m radius? *

1 ☐ Totally Agree (over 1)
2 ☐ Agree (only 1)
3 ☐ Disagree (none)

8.17 Are there public transportation stops in sight near the park? *

1 ☐ Yes, how many? ____________________________
2 ☐ No

***END OF THE QUESTIONNAIRE**

* *Reverse item*
