# Supplementary material for: Development and Validation of the Mexican Public Open Spaces Tool (MexPOS)
Source: Int J Environ Res Public Health. 2022 Jul 5;19(13):8198. doi: 10.3390/ijerph19138198 (PMC9266626; doi:10.3390/ijerph19138198)
Supplement: Supplementary file 1 [file ijerph-19-08198-s001.zip › File S3.pdf]

**Starting models (items)**	**MODEL I**		**MODEL II**		**MODEL III**		**MODEL IV (survey)**
Original questionnaire (181 items) Based on: BRAT-DO, EAPRS, and a group of experts (Appendix 1)	Advertisement and land uses<br>F1 (14)	EXPERTS TECHNIQUE<br>Date:<br>(2021/03/03)<br>Main conclusions:<br>(F7, F10, F11, F12 and F13 allocated to other factors)	Advertisement and land uses<br>F1 (7)	EXPERTS TECHNIQUE<br>Date:<br>(2021/04/05)<br>Main conclusions:<br>(F4 and F7 allocated to other factors)	Food environment and wellbeing<br>F1 (7)	EXPERTS TECHNIQUE<br>Date:<br>(2021/05/10)<br>Main conclusions:<br>General park information was included. This information came from the first version of the questionnaire (n=3 items)	General park information<br>F1 (3)
	Amenities and maintenance<br>F2 (27)		Amenities and maintenance<br>F2 (41)		Maintenance<br>F2A (20)		Food Environment and Health wellness<br>F2 (6)
	Signs and announcements<br>F3 (10)		Signs and announcements<br>F3 (13)		Amenities<br>F2B (44)		Maintenance<br>F3 (21)
	Outdoor<br>F4 (15)		Outdoor and activities<br>F4 (16)		Readability (signs announcements)<br>F3 (15)		Amenities<br>F4 (44)
	Safety and cycling<br>F5 (8)		Safety and cycling<br>F5 (22)		Safety<br>F5 (11)		Signaling<br>F5 (15)
	Pedestrians<br>F6 (11)		Pedestrians<br>F6 (10)		Accessibility<br>F6 (9)		Safety<br>F6 (11)
	Sport area maintenance<br>F7 (10)		Sensory elements<br>F8 (9)		Perceived environment<br>F8 (13)		Perceived environment<br>F7 (14)
	Sensory elements<br>F8 (9)		Streets<br>F9 (10)		Urban environment<br>F9 (9)		Urban surroundings<br>F8 (17)
	External context<br>F9 (4)						
	Streets<br>F10 (4)						
	Outdoor sport facilities<br>F11 (6)						
	Inside park characteristics<br>F12 (5)						
	Other park characteristics<br>F13 (5)						
**Total items**	128		128		128		131
