# Supplementary material for: Development and Validation of the Mexican Public Open Spaces Tool (MexPOS)
Source: Int J Environ Res Public Health. 2022 Jul 5;19(13):8198. doi: 10.3390/ijerph19138198 (PMC9266626; doi:10.3390/ijerph19138198)
Supplement: Supplementary file 1 [file ijerph-19-08198-s001.zip › Table S1-3.pdf]

Table S1. Model I

Factors	Items
**Factor 1** (α=0.43)	Do you observe uniformed park workers?
	Do you hear other sounds not mentioned above?
	Is there food and beverage advertising inside the park? *
	Does the park have parking facilities for motorbikes?
	Are there advertisements promoting cakes, cookies, or cake dough inside the park? *
	Are there signs indicating whether dogs must be on a leash or in a specific area?
	Is the land use around the park mainly commercial? *
	Are there signs restricting bicycle parking area inside the park?
	Are there guardhouses in the park?
	Have there been any security incidents so far these years?
	Based on what you observed today, is the parks attractive to you?
	In general, what is the percentage of wooded areas in the park?
	Does the park have historical, educational, or artistic features?
	Is the condition of the landscape adequate?
**Factor 2** (α=0.73)	Is the condition of the sidewalks bad? *
	Is there garbage around the park? *
	Are there empty or unpleasant buildings around the park? *
	What other areas for activities did you observe?
	Are there properties around the park in poor condition?
	Is there a fronton court inside the park?
	Are all areas of the park open to the public?
	Is there a bike path inside the park?
	Is there a football/soccer court inside the park?
	Are there any indicators of who oversees the park?
	Is there a stationary bike in the outdoor gym?
	Are there any creeks, lakes or ponds inside the park?
	Is there electric lightning inside the park?
	Are there other areas of activities not mentioned above?
	Is there a map of the park?
	Are there botanical gardens inside the park?
	Does the park have a parking lot for cars?
	Does the opening or operation of the park depend on a janitor or manager?
	Does the park have shelters?
	Is there a 5-a-side football/soccer court inside the park?
	Are there abdominal boards in the outdoor gym?
	Is the park fenced or gated?
	Are there police stations inside the park?
	Is there a shared trail around the park?
	Does the park have bathrooms?
	Are there public transportation stops in sight near the park? *
	Is the outdoor gym being used?
**Factor 3** (α=0.49)	Is there any sign of the appropriation of the park?
	Is there a skatepark area inside the park?

	Are there sings indicating other signage not mentioned above?
	Are there signs indicating where to dispose pet excrement?
	Are there posters promoting events inside the park?
	Are there garbage containers for recyclable products?
	Is there medical service inside the park?
	Are there signs indicating the park's schedule of operation?
	Are there no parking sings around the park?
	Are there bars in the outdoor gym?
**Factor 4** (α=0.43)	Is the main land use inside the park natural?
	Is there a shoulder massage machine in the outdoor gym?
	Do you hear music sounds inside the park?
	Do you hear voices sounds inside the park?
	Are there streets with sidewalks around the park?
	How much hazardous waste is visible in the park? (e.g., alcohol containers, preservatives…) *
	Does the outdoor gym have a surfboard?
	Is there any other apparatus not mentioned above inside the outdoor gym?
	Does the outdoor gym have a back massage machine?
	Is the solar panel lighting inside the park?
	Is the waist twist inside the outdoor gym?
	Are there signs indicating whether the park has equipment for rental?
	Is the outdoor gymnasium in good condition?
	Does the park have lighting?
	Are there any announcements indicating the road, route, track of higher hierarchy?
**Factor 5** (α=0.41)	Does the park have a street inside?
	Is there a bike route that leads to the park?
	Do you see graffiti around the park? *
	Is there poor street lighting around the park?
	Are the eating areas in good condition?
	In total, how much of the park could be shaded?
	Are the green areas in good condition?
	Does the park have parking facilities for bikes?
**Factor 6** (α=0.6)	Do you hear bird sound inside the park?
	Does the park have handrails?
	Is the condition of these sidewalks regular? *
	Is the condition of the sidewalks good?
	Are there streets with zebra crossings around the park?
	Is there a police presence inside the park?
	Is there a rowing machine in the outdoor gym?
	Is the condition of the sidewalks regular?
	Are people smoking inside the park? *
	Does the park have ramps to guarantee access to the park?
	Is there a ski walker in the outdoor gym?
**Factor 7** (α=0.6)	Are the seating areas in good condition?
	Are the walking paths in good condition?
	Is the playground area in good condition?

	Is there vandalism inside the park?
	Is the condition of the road/route/major trail good?
	Is there a 7 football/soccer field in the park?
	Are most of the streets around the park secondary?
	In general, is the park well maintained?
	Is the state of the places to sit good?
	Are there security cameras inside the park?
**Factor 8** (α=0.65)	Are there courts that combine soccer and basketball?
	Are most of the streets around the park tertiary?
	Do you hear water sounds inside the park?
	Are there public transport stations within a 100m radius? *
	Are most of the streets around the park primary? *
	Is there excessive noise around the park? *
	Do you see panic buttons in the park?
	Do you observe animal excrement in the park? *
	Is heavy traffic around the park? *
**Factor 9** (α=0.69)	Are there streets with traffic lights around the park?
	Are there any streets with pedestrian traffic lights around the park?
	Are there streets with safe crossings around the park?
	Is there a floating treadmill in the outdoor gym?
**Factor 10** (α=0.28)	Is there a leg press machine in the outdoor gym?
	Is there garbage in the park, other than around and/o inside the trash cans? *
	Are there pedestrian streets around the park?
	Does the park have drinking fountains?
**Factor 11** (α=0.36)	Is there a colt in the outdoor gym?
	Is there an elliptical trainer in the outdoor gym?
	Does the outdoor gym have hoops?
	According to today's experience, is the smell of the park pleasant?
	Does the outdoor gym have hoops and a waistband?
	Is there a basketball court inside the park?
**Factor 12** (α=0.06)	Is there any reference or sign indicating the name of the park?
	Is the land use around the park mainly institutional?
	Are the trees the main source of shade in the park?
	Is there any evidence of threatening people or behaviour?
	Does the outdoor gym have a multifunctional machine?
**Factor 13** (α=0.06)	Are there food and beverage stalls around the park? *
	Is there a chest press machine in the outdoor gym?
	Is there a jogging path inside the park?
	Do you hear traffic sounds inside the park? *
	Write down the number of stalls around the park*

* Reverse-coded items

Table S2. Model II

Factors	Items
**Factor 1** **Advertisement and land uses** (α=0.12)	Do you observe uniformed park workers?
	Do you hear other sounds not mentioned above?
	Is there food and beverage advertising inside the park? *
	Are there advertisements promoting cakes, cookies, or cake dough inside the park? *
	Is the land use around the park mainly commercial? *
	Based on what you observed today, is the parks attractive to you?
	Is the condition of the landscape adequate?
**Factor 2** **Amenities and maintenance** (α=0.70)	Is the land use around the park mainly institutional?
	Are there food and beverage stalls around the park? *
	Write down the number of stalls around the park. *
	Is the condition of the sidewalks bad? *
	Is there garbage around the park? *
	Are there empty or unpleasant buildings around the park? *
	What other areas for activities did you observe?
	Are there properties around the park in poor condition?
	Is there a fronton court inside the park?
	Are all areas of the park open to the public?
	Is there a football/soccer court inside the park?
	Is there a stationary bike in the outdoor gym?
	Are there other areas of activities not mentioned above?
	Is there a map of the park?
	Are there botanical gardens inside the park?
	Does the park have a parking lot for cars?
	Does the opening or operation of the park depend on a janitor or manager?
	Does the park have shelters?
	Is there a 5-a-side football/soccer court inside the park?
	Are there abdominal boards in the outdoor gym?
	Is the park fenced or gated?
	Does the park have bathrooms?
	Are there public transportation stops in sight near the park? *
	Is the outdoor gym being used?
	Is there a rowing machine in the outdoor gym?
	Is there a ski walker in the outdoor gym?
	Is there a colt in the outdoor gym?
	Is there an elliptical trainer in the outdoor gym?
	Is there a leg press machine in the outdoor gym?
	Does the outdoor gym have hoops?
	Is there a chest press machine in the outdoor gym?
	Does the outdoor gym have a multifunctional machine?
	Does the outdoor gym have hoops and a waistband?
	Are there bars in the outdoor gym?
	Is there a floating treadmill in the outdoor gym?
	Is the playground area in good condition?

	Is there a 7 football/soccer field in the park?
	Are there courts that combine soccer and basketball?
	Is there a basketball court inside the park?
	Does the park have historical, educational, or artistic features?
	Is there a skatepark area inside the park?
**Factor 3 Signs and announcements** (α=0.50)	In general, is the park well maintained?
	Is there medical service inside the park?
	Does the park have parking facilities for motorbikes?
	Is there any sign of the appropriation of the park?
	Are there sings indicating other signage not mentioned above?
	Are there signs indicating where to dispose pet excrement?
	Are there posters promoting events inside the park?
	Are there garbage containers for recyclable products?
	Are these signs indicating the park's schedule of operation?
	Are there no parking sings around the park?
	Are there signs indicating whether dogs must be on a leash or in a specific area?
	Is there any reference or sign indicating the name of the park?
	Are there any announcements indicating the road, route, track of higher hierarchy?
**Factor 4 Outdoor and activities** (α=0.45)	Are there signs indicating whether the park has equipment for rental?
	Are there any indicators of who oversees the park?
	Is the main land use inside the park natural?
	Is there a shoulder massage machine in the outdoor gym?
	Do you hear music sounds inside the park?
	Do you hear voices sounds inside the park?
	Are there streets with sidewalks around the park?
	How much hazardous waste is visible in the park? (e.g., alcohol containers, preservatives…) *
	Does the outdoor gym have a surfboard?
	Is there any other apparatus not mentioned above inside the outdoor gym?
	Does the outdoor gym have a back massage machine?
	Is the solar panel lighting inside the park?
	Is the waist twist inside the outdoor gym?
	Is the outdoor gymnasium in good condition?
	Does the park have lighting?
	Does the park have drinking fountains?
**Factor 5 Safety and cycling** (α=0.64)	Are there any creeks, lakes, or ponds inside the park?
	In general, what is the percentage of wooded areas in the park?
	Does the park have a street inside?
	Is there a bike route that leads to the park?
	Do you see graffiti around the park? *
	Is there poor street lighting in the area around the park?
	Are the eating areas in good condition?
	In total, how much of the park could be shaded?
	Are the green areas in good condition?
	Does the park have parking facilities for bikes?

	Are there security cameras inside the park?
	Do you see panic buttons in the park?
	Is there vandalism inside the park?
	Is there a police presence inside the park?
	Is there electric lightning inside the park?
	Are there guardhouses in the park?
	Are there police stations inside the park?
	Have there been any security incidents so far these years?
	Are there signs restricting bicycle parking area inside the park?
	Is the condition of the road/route/major trail good?
	Is there a jogging path inside the park?
	Is there a bike path inside the park?
**Factor 6 Pedestrians** (α=0.61)	Is there a shared trail around the park?
	Do you hear bird sound inside the park?
	Does the park have handrails?
	Is the condition of these sidewalks regular? *
	Is the condition of the sidewalks good?
	Is the condition of the sidewalks regular?
	Are people smoking inside the park? *
	Does the park have ramps to guarantee access to the park?
	Is there any evidence of threatening people or behaviour?
	Are the seating areas in good condition?
*The items from **Factor 7** were allocated to other factors in which they theoretically fit better as suggested by an expert group.*	
**Factor 8 Sensory elements** (α=0.61)	Is the state of the places to sit good?
	Are the walking paths in good condition?
	Are there public transport stations within a 100m radius? *
	Do you hear water sounds inside the park?
	Is there excessive noise around the park? *
	Is heavy traffic around the park? *
	Do you observe animal excrement in the park? *
	According to today's experience, is the smell of the park pleasant?
	Is there garbage in the park, other than around and/o inside the trash cans? *
**Factor 9 Streets** (α=0.63)	Are the trees the main source of shade in the park?
	Do you hear traffic sounds inside the park? *
	Are most of the streets around the park primary? *
	Are there any streets with pedestrian traffic lights around the park?
	Are there streets with traffic lights around the park?
	Are there streets with safe crossings around the park?
	Are most of the streets around the park secondary?
	Are most of the streets around the park tertiary?
	Are there streets with zebra crossings around the park?
	Are there pedestrian streets around the park?
*The items from **Factor 10, 11, 12 & 13** were allocated to other factors in which they theoretically fit better as suggested by an expert group.*	

* Reverse-coded items

Table S3. Model III

Factor	Items
**Factor 1 Food environment and wellbeing** (α=0.10)	Is there food and beverage advertising inside the park? *
	Are there advertisements promoting cakes, cookies, or cake dough inside the park? *
	Is the land use around the park mainly commercial? *
	Is the condition of the landscape adequate?
	Is the land use around the park mainly institutional?
	Are there food and beverage stalls around the park? *
	Write down the number of stalls around the park. *
**Factor 2A Maintenance** (α=0.78)	Do you observe animal excrement in the park? *
	Is the condition of the road/route/major trail good?
	Are the eating areas in good condition?
	Do you see graffiti around the park? *
	How much hazardous waste is visible in the park? (e.g., alcohol containers, preservatives…) *
	Is the outdoor gymnasium in good condition?
	Is the condition of the sidewalks bad? *
	Is there garbage around the park? *
	Are there empty or unpleasant buildings around the park? *
	Are there properties around the park in poor condition?
	Does the opening or operation of the park depend on a janitor or manager?
	Is the playground area in good condition?
	Are the green areas in good condition?
	Is the condition of the sidewalks good?
	Is the condition of the sidewalks regular?
	Are the seating areas in good condition?
	Is the state of the places to sit good?
	Is there garbage in the park, other than around and/o inside the trash cans? *
	Are the walking paths in good condition?
	In general, is the park well maintained?
**Factor 2B Amenities** (α=0.72)	Does the park have parking facilities for bikes?
	Is there a bike route that leads to the park?
	Is there electric lightning inside the park?
	Is there a shoulder massage machine in the outdoor gym?
	What other areas for activities did you observe?
	Is there a fronton court inside the park?
	Are all areas of the park open to the public?
	Is there a football/soccer court inside the park?
	Is there a stationary bike in the outdoor gym?
	Are there other areas of activities not mentioned above?
	Are there botanical gardens inside the park?

	Does the park have a parking lot for cars?
	Does the park have shelters?
	Is there a 5-a-side football/soccer court inside the park?
	Are there abdominal boards in the outdoor gym?
	Is the park fenced or gated?
	Does the park have bathrooms?
	Is the outdoor gym being used?
	Is there a rowing machine in the outdoor gym?
	Is there a ski walker in the outdoor gym?
	Is there a colt in the outdoor gym?
	Is there an elliptical trainer in the outdoor gym?
	Is there a leg press machine in the outdoor gym?
	Does the outdoor gym have hoops?
	Is there a chest press machine in the outdoor gym?
	Does the outdoor gym have a multifunctional machine?
	Does the outdoor gym have hoops and a waistband?
	Are there bars in the outdoor gym?
	Is there a floating treadmill in the outdoor gym?
	Is there a 7 football/soccer field in the park?
	Are there courts that combine soccer and basketball?
	Is there a basketball court inside the park?
	Does the outdoor gym have a surfboard?
	Is there any other apparatus not mentioned above inside the outdoor gym?
	Does the outdoor gym have a back massage machine?
	Is the solar panel lighting inside the park?
	Is the waist twist inside the outdoor gym?
	Does the park have lighting?
	Does the park have drinking fountains?
	Are there any creeks, lakes, or ponds inside the park?
	Is there a jogging path inside the park?
	Is there a bike path inside the park?
	Is there a skatepark area inside the park?
	Does the park have parking facilities for motorbikes?
**Factor 3 Readability (signs and announcements)** (α=0.58)	Are there signs restriction bicycle parking area inside the park?
	Does the park have historical, educational, or artistic features?
	Is there a map of the park?
	Is there any sign of the appropriation of the park?
	Are there sings indicating other signage not mentioned above?
	Are there signs indicating where to dispose pet excrement?
	Are there posters promoting events inside the park?
	Are there garbage containers for recyclable products?
	Are these signs indicating the park's schedule of operation?
	Are there no parking sings around the park?
	Are there signs indicating whether dogs must be on a leash or in a specific area?
	Is there any reference or sign indicating the name of the park?

	Are there any announcements indicating the road, route, track of higher hierarchy?
	Are there signs indicating whether the park has equipment for rental?
	Are there any indicators of who oversees the park?
*The items from **Factor 4** were allocated to other factors in which they theoretically fit better as suggested by an expert group.*	
**Factor 5** **Safety** (α=0.48)	Is there medical service inside the park?
	Is there poor street lighting in the area around the park?
	Do you observe uniformed park workers?
	Are there security cameras inside the park?
	Do you see panic buttons in the park?
	Is there vandalism inside the park?
	Is there a police presence inside the park?
	Are there guardhouses in the park?
	Are there police stations inside the park?
	Is there any evidence of threatening people or behaviour?
	Have there been any security incidents so far these years?
**Factor 6** **Accessibility** (α=0.42)	Are there public transport stations within a 100m radius? *
	Does the park have a street inside?
	Are there public transportation stops in sight near the park? *
	Are there streets with sidewalks around the park?
	Is there a shared trail around the park?
	Does the park have handrails?
	Is the condition of these sidewalks regular? *
	Are people smoking inside the park? *
	Does the park have ramps to guarantee access to the park?
**Factor 8** **Perceived environment** (α=0.64)	Do you hear bird sound inside the park?
	In total, how much of the park could be shaded?
	In general, what is the percentage of wooded areas in the park?
	Do you hear music sounds inside the park?
	Do you hear voices sounds inside the park?
	Do you hear other sounds not mentioned above?
	Based on what you observed today, is the parks attractive to you?
	Do you hear water sounds inside the park?
	Is there excessive noise around the park? *
	Is heavy traffic around the park? *
	According to today's experience, is the smell of the park pleasant?
	Are the trees the main source of shade in the park?
	Do you hear traffic sounds inside the park? *
**Factor 9** **Urban environment** (α=0.55)	Is the main land use inside the park natural?
	Are most of the streets around the park primary? *
	Are there any streets with pedestrian traffic lights around the park?
	Are there streets with traffic lights around the park?
	Are there streets with safe crossings around the park?
	Are most of the streets around the park secondary?
	Are most of the streets around the park tertiary?
	Are there streets with zebra crossings around the park?

	Are there pedestrian streets around the park?

* Reverse-coded items
